# Guided Bone Regeneration in the Posterior Mandible Using a Resorbable Metal Magnesium Membrane and Fixation Screws: A Case Report

**DOI:** 10.1155/crid/2659893

**Published:** 2024-12-13

**Authors:** Thomas Franke, Tadas Korzinskas

**Affiliations:** ^1^Privatärztliches Zentrum für biologische Mund-, Kiefer-, Gesichtschirurgie und Zahnmedizin, Stuttgarter Platz 1, Charlottenburg 10627, Berlin, Germany; ^2^Department of Prosthodontics, Geriatric Dentistry and Craniomandibular Disorder, Charité—Universitätsmedizin Berlin, Aßmannshauser Straße 4–6 14197, Berlin, Germany

**Keywords:** biodegradable, bone regeneration, magnesium fixation screws, magnesium membrane, staged GBR

## Abstract

**Background:** Due to bone loss, implant placement in the posterior mandible is often impossible without prior augentative procedures. The reconstruction of bone defects with horizontal and vertical components using particulated bone grafts requires the placement of a mechanically stable structure for stabilization of the grafting material. Although titanium-reinforced membranes and titanium meshes have been shown to be effective in this indication, the necessity of their removal, often in a separate surgical procedure, is seen as a disadvantage. Since the introduction of a new resorbable magnesium metal membrane and fixation screw, a mechanically stable and resorbable system might provide an alternative option for guided bone regeneration (GBR) in the posterior mandible.

**Case Presentation:** A 61-year-old patient was presented with large edentulous areas in all posterior regions and requested fixed dentures in Areas 34–36. Tooth 33 was extracted and treated with an immediate implantation of a ceramic implant, whereas Positions 34–36 were treated with a two-stage approach. The site was augmented horizontally, with a slight vertical component using autologous and allogenic bone and a new completely resorbable magnesium metal membrane and fixation screw. During the initial healing period, the patient reported a tingling sensation at the site of the augmentation. This is an observation that is specific to the magnesium products and is potentially caused by the release of hydrogen gas as the metal degrades and is resorbed. Upon re-entry at 3 months, it was clinically observed that there was a very dense and vascularized bone that was sufficient for placing two 5.5 × 10 mm ceramic dental implants.

**Conclusion:** A completely resorbable magnesium membrane and fixation screw were able to support the bony regeneration in a large GBR situation in the posterior mandible. Due to the use of a new material for GBR, different clinical observations were made compared to the standard material choices.

## 1. Introduction

For successful prosthetic implant placement, sufficient bone height and width are required. This poses a challenge for the treatment of patients with tooth loss in the posterior mandible, as this region is quickly accompanied by bone loss, which is contributed to by the absence of physiological loading [1]. Therefore, a bone augmentation procedure is often required prior to dental implant placement. Augmentation of the posterior mandible is challenging due to limited accessibility alongside anatomical structures, such as the presence of the inferior alveolar nerve, the surrounding soft tissue and muscle, and a poorer blood supply [2]. Another treatment option is to use short implants, which provide an immediate approach that can be used in atrophic but pristine alveolar ridges. However, there is no clear evidence for choosing one methodology over another [3].

Guided bone regeneration (GBR) has been proven to provide successful and predictable results in the posterior mandible [4–6]. In a recent systematic review on vertical augmentation of the posterior mandible comparing onlay block grafts and GBR, the vertical bone gain was on average higher for GBR grafts (4.7 mm) compared with block-only grafts (4.05 mm), with follow-up periods ranging between 6 and 38 months [6]. At the 1-year timepoint, there was a greater peri-implant bone loss in the onlay bone graft group. The highest gains in bone height within the GBR group were reported when nonresorbable PTFE membranes were used. There was also a lower rate of resorption in the GBR group, as well as a lower complication rate (20% vs. 11.6%). The most commonly reported complication for both groups was wound dehiscence.

Different materials are available for barrier membranes used in GBR procedures [7]. The most commonly used are collagen membranes, which offer excellent biocompatibility and are easy to handle. However, collagen membranes often have poor mechanical properties and can have a fast degradation rate, which makes them unsuitable for treating large defects with vertical components [8, 9]. An alternative option is the use of nonresorbable titanium-reinforced membranes and meshes that provide more mechanical stability and volume maintenance to support the bony regeneration within the defect [10]. However, as these are nonresorbable materials, they must be extracted at a later timepoint, increasing the morbidity for the patient [11]. Additionally, healing complications of titanium meshes can lead to the partial or complete failure of the bone augmentation [12].

Magnesium metal provides a possible alternative material choice for GBR as it is completely resorbable. As magnesium metal degrades, it releases magnesium ions (Mg^2+^) that are naturally found within the body and are present in almost every cell [13]. These are involved in many important processes within the body and have been reported to be beneficial for the formation and health of bones [14], as well as promoting angiogenesis [15]. Previous research has investigated the potential of magnesium implants for oral and maxillofacial applications such as fracture fixation systems, bone grafting, and the modification of implant surfaces [16, 17]. A newly developed pure magnesium membrane and magnesium alloy fixation screw provide an alternative option of a mechanically strong yet completely resorbable regenerative system [13–19].

The mechanical stability of the magnesium membrane enables it to protect the defect from collapse and resist the external pressure applied by the overlying soft tissue onto the graft, which can reduce the overall bone volume attained [20]. A comparison of the maximum tensile test of the magnesium membrane has been reported as 183.0 ± 10.7 MPa [21] compared to collagen which has reported values ranging between 4.8 and 22.5 MPa [22, 23]. The magnesium alloy fixation screw needs to be strong enough to retain the position of the membrane during the critical healing period, thereby ensuring that the barrier capabilities of the membrane are fulfilled. Previously published tests comparing bone anchorage and resistance to shear forces for the magnesium alloy fixation screw and a commercial resorbable polymeric alternative reported a ninefold and 3.5-fold improvement, respectively, for the magnesium device [18]. A previous in vivo study has demonstrated that the magnesium alloy fixation screws are just as efficient as titanium screws for GBR applications [24].

Further preclinical studies have reported the mechanical stability of the membrane and the fixation screws and their superior mechanical strength over alternative resorbable options [18, 21]. Furthermore, as they are completely resorbable and degrade into nontoxic, biocompatible byproducts that are easily resorbed by the body (Zheng, Gu, and Witte [25]), their use simplifies the overall procedure, as the devices do not need to be surgically removed after the healing period. In vivo animal studies in a beagle dog model have reported the degradation time for the membrane as between 2 and 4 months [19] and for the fixation screws as approximately 1 year [19].

Recent clinical cases published on the magnesium membrane and fixation screw have demonstrated their potential for regenerating defects with both horizontal and vertical components [26–32]; however, none of the cases have yet reported on their application for the treatment of medium–large defects spanning multiple positions in the posterior mandible. Especially those where it is recommended to use a nonresorbable barrier membrane (Class h3i, v2i, v3i, c2i, c3i, h3e, and c3e according to the Cologne Classification of Alveolar Ridge Defects by the European Association of Dental Implantologists (BDIZ EDI)). The aim of this case report is to demonstrate the potential for this completely resorbable metal system for GBR in the posterior mandible in a two-stage augmentation procedure.

## 2. Case Presentation

A 61-year-old patient in generally good health presented herself in the practice in May 2022. Her incomplete dentition had large edentulous areas in all posterior regions, which were provided with bridges that were as large as up to four units in size (fourth quadrant). She had the explicit desire to provide the edentulous Areas 34–36 with fixed dentures. A plan was made to treat the third quadrant first, followed by quadrant four at a later date.

**Figure 1:**
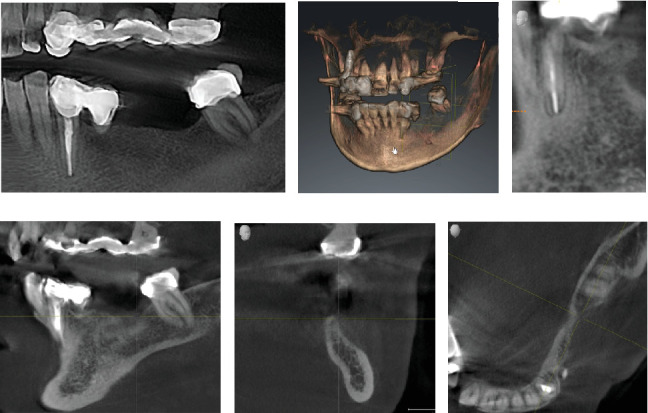
(a) Initial OPG showing a large defect between Sites 34 and 37, (b) CBCT of defect site, (c) CBCT image of the apical lesion in Site 33, and (d–f) CBCT perspectives of defect site demonstrating severe bone loss.

She was also aware of an existing problem regarding the root canal–treated Tooth 33. This tooth had been restored with a single tooth-supported prosthesis and a cantilever that was highly overloaded, which had led to a widened periodontal ligament (PDL) and resulted in an increased degree of loosening. Nonaxial occlusal forces and torque caused by the cantilever were causing severe pain to the patient. In addition, the tooth showed an incipient lesion apically (Figure 1(c)).

Cone beam computed tomography (CBCT) showed very strong generalized horizontal bone resorption, especially in the edentulous molar regions of all quadrants (Figure 1).

The patient was recommended to have Tooth 33 extracted as it could no longer benefit from endodontic treatment and had marked mobility. It was also the cause of severe pain for the patient. Removal of the cantilever might have alleviated the symptoms; however, the poor condition of the tooth and overinstrumented root canal necessitated the extraction of the tooth, especially due to its positioning next to the planned augmentation site. After extraction, Position 33 would subsequently receive an immediate placement of a ceramic implant. A two-stage approach was planned for Positions 34–36: a comprehensive augmentation of the site, followed by the placement of ceramic implants in Positions 35 and 36 in a second-stage surgery. An overview of the surgical plan is shown in Table 1.

A lymphocyte transformation test was performed preoperatively and supported the material choices. The test is used to determine hypersensitivity towards different materials and drugs by measuring the proliferation of T cells in vitro and monitoring for signs of sensitization reactions [33].

Preoperatively, the patient was placed with an intravenous line, through which a blood sample was taken for the collection of plasma rich in growth factors (PRGF). A multivitamin infusion, consisting of vitamin C, magnesium, and bicarbonate, was administered to promote soft tissue healing [34]. The operation was performed under sedation (midazolam 3 mL IV) and local anesthesia by infiltration of a vasoconstrictor-containing anesthetic with epinephrine 1:200,000 in Positions 34–36.

Under perioperative antibiotic prophylaxis (clindamycin 600 IV), the crestal incision of the alveolar process was performed. In this case, the mucous membrane and submucosa as well as the periosteum were severed. The periosteum was separated away from the bone to the vestibular and lingual directions using a periosteal elevator.

**Figure 2:**
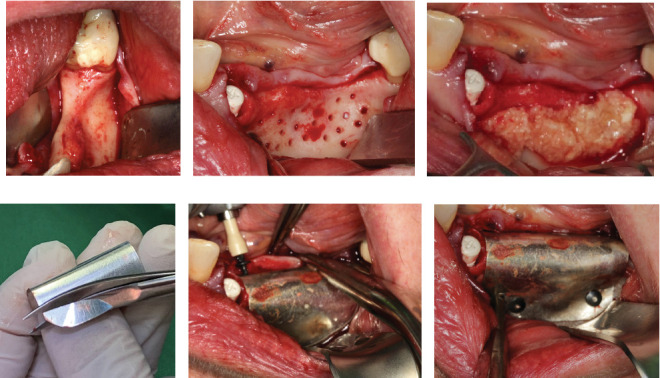
Augmentation procedure using the magnesium membrane. (a) Exposure of the defect showing the initial situation. (b) The cortical bone is perforated in multiple places to improve blood flow into the augmentation site. (c) The defect site is augmented with a mix of allograft, autogenous bone, and liquid PRGF in a 1:1:1 ratio, after which additional PRGF is positioned on top. (d) The magnesium membrane is cut and bent into shape prior to its placement over the defect. (e, f) The membrane is secured on both the lingual and the buccal sides using magnesium fixation screws.

After Tooth 33 had been extracted, the extraction socket was disinfected with ozone in a gaseous form, which has been reported to have strong antimicrobial activity (Eick, Tigan, and Sculean [35]). The ozone probe was placed within the socket for 1 min. This was followed by the immediate insertion of a ceramic implant (XT RB 4.2 × 12 mm, Zeramex, Switzerland) into the extraction socket alongside allogeneic graft granules (Puros, ZimVie, United States), autologous bone, and PRGF in an approximate 1:1:1 ratio. PRGF was incorporated into the graft due to a general trend of its inclusion improving the clinical outcome for bone augmentation (Blanco, Caramês, and Quirynen). Additionally, its inclusion into the graft material creates a sticky graft that improves handling and application [36]). Thereafter, multiple perforations of the alveolar process were performed to a depth of about 3 mm to improve the vascularization of the graft (Figure 2(b)).

The alveolar ridge was augmented using allogeneic (Puros, ZimVie, United States) and autologous bone graft granules, retrieved from the third quadrant using a Safescraper, and mixed together with liquid PRGF to form a sticky bone in an approximate 1:1:1 ratio (Figure 2(c)). A PRGF membrane was then positioned on top of the augmentation material.

**Figure 3:**
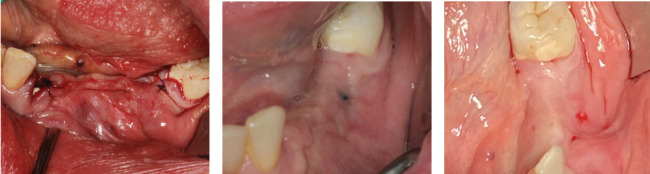
Soft tissue healing over the augmentation. (a) Wound closure using a continuous seam stitch; (b) wound control, 3 weeks post-op; (c) 3 months post-op, prior to re-entry and dental implant placement.

**Figure 4:**
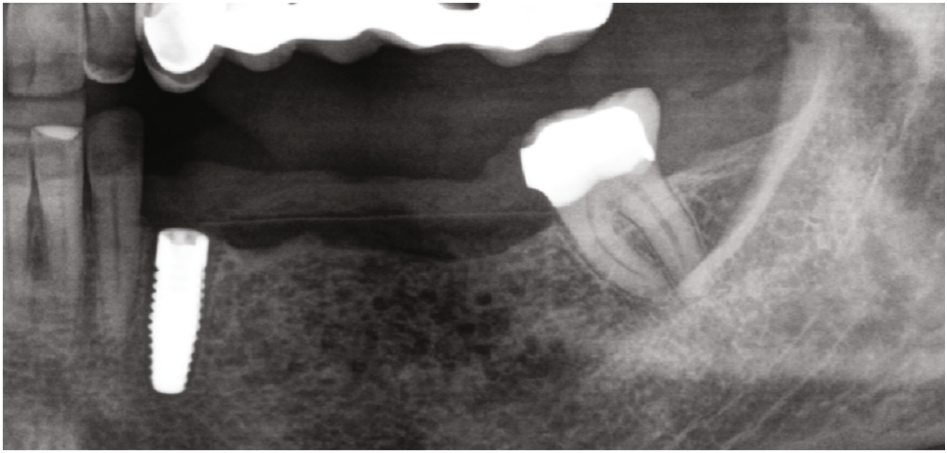
OPG taken immediately after the augmentation procedure.

Subsequently, a pure magnesium membrane (NOVAMag membrane, Botiss GmbH, Germany) was cut and then bent to shape prior to placement over the graft (Figure 2(d)). The magnesium membrane was fixed into position on both the vestibular and lingual sides using magnesium alloy fixation screws (NOVAMag fixation screw XS, Botiss Biomaterials GmbH, Germany) (Figures 2(e) and 2(f)). Prior to the insertion of the magnesium alloy fixation screws, pilot holes were drilled through the membrane and into the patient's native bone. To prepare the mucoperiosteal flap for tension-free wound closure, the periosteum was cut, and a continuous suture was used for closure (Figure 3(a)). An OPG was taken immediately after the augmentation (Figure 4). The magnesium membrane is only visible where it is positioned perpendicular to the x-ray.

The postsurgical protocol included the intravenous administration of a painkiller (1000 mg Novalgin, Sanofi, Germany) and the application of a cooling mask (Hilotherm GmbH, Germany) for 1.5 h to reduce swelling, bruising, inflammation, and pain (Glass, Waterhouse, and Shakib [37]). For the first 7 days post-op, the patient was prescribed 600 mg/day of ibuprofen to be taken ad libitum. The patient was directed to have a liquid food intake for the first 14 days post-op.

During the postsurgical follow-up, the patient reported a moderate level of pain locally at the surgical site. This was in combination with swelling and a slight redness.

No additional treatment measures were prescribed for the patient, and the pain, swelling, and redness were resolved within 1 week.

Between 1 and 2 weeks after surgery, the patient reported a prickly feeling at the surgical site which persisted for 1–2 weeks before resolving. This is potentially related to the release of hydrogen gas during the degradation of the magnesium metal [24].

A thick keratinized soft tissue formed over the treatment site prior to re-entry (Figure 3(c)). At 3 weeks, the head of the magnesium fixation screw was visible through the soft tissue (Figure 3(b)). However, by 3 months post-op, prior to re-entry, there was a complete covering with thick keratinized tissue over the augmented site, and the screwhead was no longer visible (Figure 3(c)).

**Figure 5:**
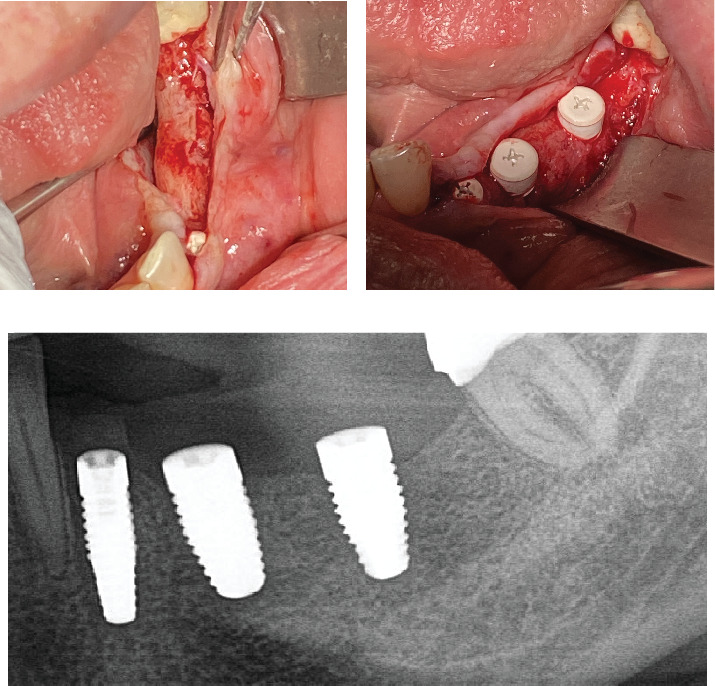
(a) Re-entry after 3 months; (b) placement of two 5.5-mm-diameter ceramic implants in Positions 35 and 36; (c) control OPG taken after implant placement.

Re-entry was performed after a 3-month healing phase. Upon opening the flap, densely mineralized bone growth was clinically observed with a significant horizontal volume increase (Figure 3(a)). Due to the bone quantity and quality, implants could be positioned at Sites 35 and 36. Under the same clinical conditions as in the first procedure, two ceramic implants (XT WB 5.5 × 10 mm, Zeramex, Switzerland) were successfully implanted with an insertion torque of 35 Ncm, as is the protocol as stated by the manufacturer (Figures 5(b) and 5(c)).

The implants were submerged for a healing time of 4 months prior to loading.

**Figure 6:**
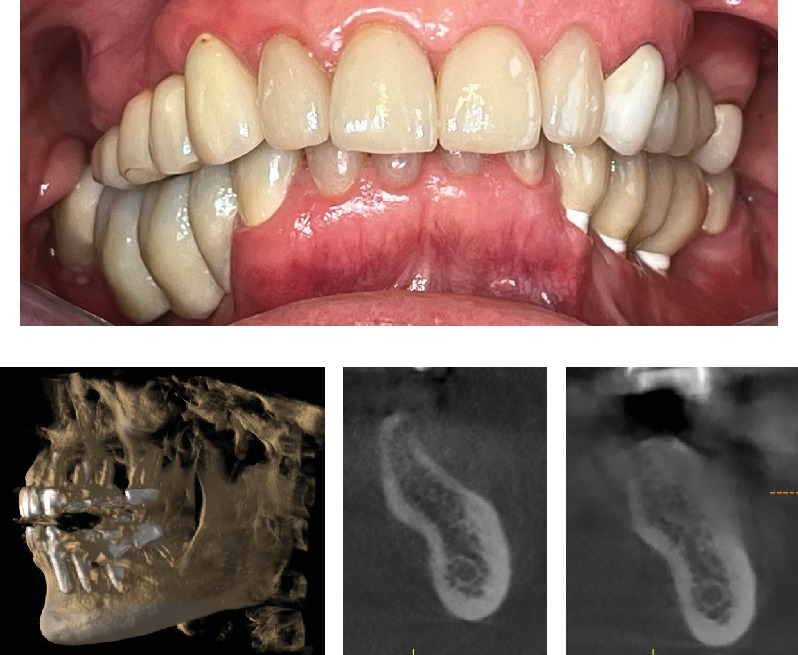
One-year follow-up after implant placement. (a) Frontal view clinical photograph, (b) CBCT showing distortion due to the presence of artifacts caused by the ceramic implants, (c) CBCT cross-sectional view of defect site prior to augmentation, and (d) CBCT cross-sectional view of defect at 1-year follow-up.

A 1-year follow-up of the case demonstrates stability to the augmented bone. A loss of soft tissue surrounding the implants was observed (Figure 6(a)); however, the patient refused further treatment. Due to the symmetrically low soft tissue line on both sides of the lower jaw, it is possible that the gingival recession could be attributed to genetic or behavioral reasons [38]. CBCT taken before augmentation and after 1 year with implant placement show the extent of the regenerated bone (Figures 6(c) and 6(d)). Unfortunately, due to the artifacts produced by ceramic implants (Warren, Vaddi, and Tadinada [39]), the image quality is poor and therefore does not allow a quantitative evaluation of the regenerated bone.

## 3. Discussion

Bone regeneration in the posterior mandible presents a challenge, both physiologically and technically. After tooth loss, the region is prone to bone resorption due to a lack of loading. For implant placement, there are two available methodologies: the use of short implants or performing a two-stage surgery.

The use of short implants has the benefit of a one-stage surgery, and in the posterior mandible, the data appears to provide comparable outcomes as those associated with normal-sized implants [3]. Nevertheless, the overall data, especially long-term outcomes, are quite limited for the use of short-length implants [3, 40]. Their use in the posterior maxilla is also reported to have a worse outcome than normal-length implants [40].

Therefore, prior to implant placement, there is usually the necessity to augment the bone. Different techniques are available for increasing the bone volume, such as the inlay technique [41] or ridge splitting [42]; however, these techniques are very technically advanced. To achieve bone volume gain in both vertical and horizontal directions, GBR is often performed using nonresorbable titanium-reinforced membranes or titanium meshes (Robert, Aloy-Prósper, and Arias-Herrera [6]). Titanium meshes or titanium-reinforced membranes can achieve substantial bone gain. However, their application is also technique-sensitive and requires expert surgeons for their successful application and removal after the healing period, especially for titanium meshes [43].

Although typical resorbable membranes can be used, composed of collagen, it was found in a systematic review and meta-analysis performed by Urban et al. [44] that resorbable membranes used for vertical augmentation without additional mechanical support could collapse, ultimately reducing the bone volume gained. Due to the physical requirements, there was also an increased complication rate associated with resorbable membranes (~23%) in comparison to nonresorbable membranes (~7%).

Therefore, due to the limitations of the most commonly chosen materials, the magnesium membrane and fixation screws present an opportunity to provide the necessary mechanical support for bony regeneration in defects with both horizontal and vertical components, but without the requirement for their extraction after the healing period. The extent to which magnesium devices can achieve vertical gain is yet to be fully established. In the presented case, the use of a magnesium membrane and fixation screw has been successfully applied for GBR in the atrophic posterior mandible. Used together, they create an entirely resorbable system, negating the need to create a large flap during re-entry for material retrieval, reducing overall patient morbidity.

The case demonstrates that in instances where there is a severely atrophied bone situation of the lower jaw, a considerable horizontal bone gain can be achieved using the magnesium membrane and fixation screws in combination with an allogenic and autologous bone graft. After a healing period of 3 months, during re-entry, highly vascularized and dense bone was clinically observed. This is due to the mechanical support provided by the magnesium metal during the initial healing period [18, 21], which provided space for bony ingrowth and is an important requirement for GBR [45]. Upon re-entry, there were no visible remnants of the magnesium membrane observed clinically, indicating its complete resorption. Additionally, there were no observations of the magnesium devices observed via the CBCT data prior to re-entry. However, this was expected, as although magnesium metal is visible via CBCT data and produces fewer artifacts in comparison to steel and titanium [46], due to the initial size of the membrane with a 140 *μ*m thickness, it is difficult to view, even immediately after implantation.

Therefore, despite the resorbable properties of the magnesium membrane and fixation screw, their mechanical support was sufficient for optimal bone regeneration. Due to the bone quality and volume that was achieved, two 5.5 × 10-mm-ceramic dental implants could be inserted at the time of re-entry (3 months).

Depending on the bone graft material used, re-entry can vary between > 3.5 months for allografts [47] and 4–9 months for xenografts [48]. Similar re-entry times have also been used in previously published cases using the magnesium membrane [26–31]. However, from the author's own clinical experience using the magnesium membrane, the quality and quantity of bone achieved when using the magnesium membrane alongside allograft bone enable the possibility of a faster re-entry. This was reconfirmed with the reported case, as at the time of re-entry at 3 months, two large-diameter implants could be placed securely in the augmented bone using 35 Ncm of torque, as is the standard protocol for the chosen implants.

The mechanical support offered by the magnesium membrane has previously been demonstrated for other indications. Frosecchi [30] reported the use of the magnesium membrane combined with a collagen membrane to create a fully resorbable reinforced membrane to successfully augment both vertically and horizontally in the anterior region of the maxilla. Elad et al. [29] used the membrane to bridge singular tooth defects with compromised sockets for immediate implant placement. In some instances, Elad et al. bent the membrane into a double layer to increase the mechanical strength of the membrane. However, in the GBR situation presented in the current case, it was not necessary to bend the membrane into a double layer, and the mechanical strength was sufficient to retain the shape and volume of the augmentation.

During the initial healing, there were several clinical observations that were associated with the new material, which were not reported by the other authors. During the first week after surgery, the patient reported pain, which was slightly stronger and lasted for longer than usually expected after a surgery involving, for example, a collagen membrane. In this case, a standard postsurgical pain management regiment was prescribed. The pain level reported by the patient could be related to the patient's own pain threshold; however, it has also been noted by the authors in other nonreported cases where the magnesium membrane has been used. Therefore, for subsequent cases, it may be beneficial to prescribe additional pain medication for the first week after surgery as a standardized part of the treatment plan for patients treated with the magnesium membrane and fixation screw, to preempt any patient discomfort. Other clinical observations during the early healing period, within the first week after surgery, included a slight swelling and redness, which resolved by themselves.

Another observation that was unique to the magnesium devices was that the patient reported a tingling feeling at the surgical site. This could be related to the release of hydrogen gas, which is a byproduct of magnesium as it degrades [49]. This phenomenon of hydrogen gas release has previously been reported for orthopedic screws made from magnesium, where it caused the creation of gas cavities but had no long-term negative effects on new bone growth [50–52]. This was also reported by Rider et al. [24] in an in vivo study performed using the magnesium membrane to treat GBR defects in beagle dogs. In the in vivo study, the release of the hydrogen gas presented itself as either a small swelling or a slight elevation of the soft tissue before it was resorbed. Importantly, as was concurrent with the previously reported studies, there was no negative effect of hydrogen gas production on the formation of new bone.

Nevertheless, additional swelling of the soft tissue might increase the risk of wound dehiscence. This is yet to be reported on regarding cases with the magnesium membrane [26–31] but could be anticipated due to the use of a rigid membrane interfacing with the soft tissue, which is known to increase the likelihood of membrane exposure [53]. However, an in vitro test on the magnesium membrane demonstrated a good adhesion of human gingival fibroblasts to the degrading membrane, as well as their migration over its surface [54].

As a summary of the author's own experience reported in this case and in other nonreported cases using the magnesium membrane, several observations have been made. These include additional pain during the early healing that could be alleviated by actively prescribing additional pain medication during the first week post-op; a tingling sensation at the surgical site might be experienced by the patient, potentially caused by the release of hydrogen gas as the magnesium metal degrades; a very dense and vascularized bone is observed clinically upon re-entry; and the possibility to shorten re-entry times, thereby speeding up patient treatments due to the excellent regenerative bone results. As these observations are only reported in this case report, additional clinical data is required to verify their association with the magnesium membrane. For example, additional clinical data could be collected via a clinical study comparing postoperative observations during the healing period between patients undergoing GBR treatment using different GBR membranes, with both resorbable and nonresorbable options. Additionally, future studies should consider the histological analysis of trephine samples taken from the augmentation to determine if there is a comparative difference in bone quality and vascularization between the use of different barrier membranes.

## 4. Conclusions

Overall, the presented case demonstrates that the potential for the magnesium membrane and fixation screw is very promising for applications of GBR with large horizontal augmentations with vertical components. Bone volume is maintained due to the rigidity of the membrane providing space for the ingrowth of the bone. It was observed clinically that there was also very high quality to the regenerated bone, as at re-entry after 3 months, the bone was very dense and vascularized. Due to the material properties of magnesium and the byproducts of its degradation, the clinical observations during the healing period were slightly different from those normally experienced with other resorbable membranes such as collagen. Due to the limitation that these observations are reported as a singular case, further clinical data are required in order to assess the full potential of this new material class in regenerative dentistry to determine how consistently these observations occur, as well as determine the limitations to the size of the defect that can be treated. Additionally, the possibility for vertical augmentation needs to be further explored in order to determine when and where the magnesium membrane can be used as an alternative to reinforced membranes or titanium meshes.

## Figures and Tables

**Table 1:** Overview of surgical plan.

**Age**	**Gender**	**Area**	**Surgery**	**Use of magnesium membrane and fixation screw**	**Graft material**
61	Female	33	Tooth extraction with immediate implant placement	No	Autologous bone, allogenic bone, and PRGF in a 1:1:1 ratio
		34–36	Two-stage GBR surgery	Yes	Autologous bone, allogenic bone, and PRGF in a 1:1:1 ratio

## Data Availability

All data are included in this manuscript.
